# Racial Disparity and Trend of Food Scarcity Amid COVID-19 Pandemic in the United States

**DOI:** 10.7759/cureus.33232

**Published:** 2023-01-01

**Authors:** Sae X Morita, Hirotaka Kato

**Affiliations:** 1 Internal Medicine, St. Barnabas Hospital Health System, New York, USA; 2 Internal Medicine, University of Kentucky, Lexington, USA

**Keywords:** national trend, health disparity, race inequities, covid-19 pandemic, food insecurity

## Abstract

An increase in households with food insecurity has been reported during the COVID-19 pandemic, but the trend of food insecurity during the pandemic remains unclear. Using Household Pulse Survey (HPS) data over 34 weeks from June 2020 to September 2021 (nationally representative samples of US adults in the households from the US Census Bureau), we examined racial disparity and trends of food scarcity amid the COVID-19 pandemic. The time series plots illustrated that the food scarcity rate was incremental until December 2020 and began improving thereafter across all racial groups. Such improvements in food scarcity were accompanied by the rise in regular income rates while the use of food assistance programs, unemployment insurance, and stimulus payments remained unchanged or reduced. As the US economy recovered, the gaps in food scarcity rates also narrowed between Black/Hispanic and White households.

## Introduction

Food insecurity, defined as the limited or uncertain ability to acquire adequate food for one or more household members, is a part of social determinants of health (SDOH) and is associated with various health conditions such as obesity, diabetes mellitus, hypertension, and mental health [1,2]. According to a recent US Department of Agriculture report, in 2019, about 10.5% of US households (13.7 million households) experienced food insecurity at least part of the time, including 2.4 million households with children [3].

An increase in households with food insecurity has been reported during the COVID-19 pandemic [4]. According to projected food insecurity prevalence for 2020 and 2021 released by Feeding America, a US-based nonprofit network of more than 200 food banks that feed more than 46 million people, food insufficiency prevalence increased from 8% prior to 13 March 2020 to 10% on April and May 2020, and food insecurity was projected to increase from 11% in 2019 to 14% in 2020 [5]. Furthermore, it was reported that the financial hardship due to the pandemic disproportionately affected Black and Hispanic, and lower-income households and that those populations had a higher risk of food insufficiency [6]. Regarding the effect of food insecurity during the pandemic, several early surveys reported that the worsened food insecurity during the pandemic has adversely affected mental health [7,8]. For instance, it was reported that greater food insecurity was associated with a dose-response relationship with all psychological distress outcomes including depression, anxiety, stress, and COVID-19-specific worries [8]. However, no previous studies examined the trend of food insecurity by race throughout the pandemic, and how social benefits such as the food assistance program and COVID-19 stimulus payments mitigate food insecurity remains unclear.

In this preliminary study, we aimed to explore the trend of food insecurity among races and the source of food during the pandemic.

## Materials and methods

Dataset and study design

We conducted an exploratory data analysis of the Household Pulse Survey (HPS), publicly available from the US Census Bureau. HPS is a biweekly survey of nationally representative samples of adults in households to assess the socioeconomic and health impacts due to the COVID-19 pandemic. HPS used several strategies to sample nationally and racially representative US households. The sample sizes were the same across all sampling areas (divided into 66 areas and 15 metro areas) to help estimate precision. Survey weights are designed to adjust for possible differences among nonrespondents. The US Census Bureau defines “food scarcity” as respondents who reported “sometimes” or “often” not enough to eat in the last seven days. We also defined “at-risk of food scarcity” as respondents who reported having “enough food, but not always the kinds wanted.” In HPS, race categories were defined as Hispanic or Latino of any race, Black alone, White alone, Asian alone, and two or more races/other racial minorities. HPS survey weights [KH1] were applied to obtain the national estimates of US adults.

Statistical analyses

We created and inspected time series plots of the following national estimates over 34 weeks from June 2020 to September 2021: proportion of (1) food scarcity, stratified by race as well as (2) sources of money to buy food (i.e., regular income from work, unemployment insurance, COVID-19 stimulus payments, food assistance programs) and (3) experienced/expected unemployment in four weeks. 

## Results

**Figure 1:**
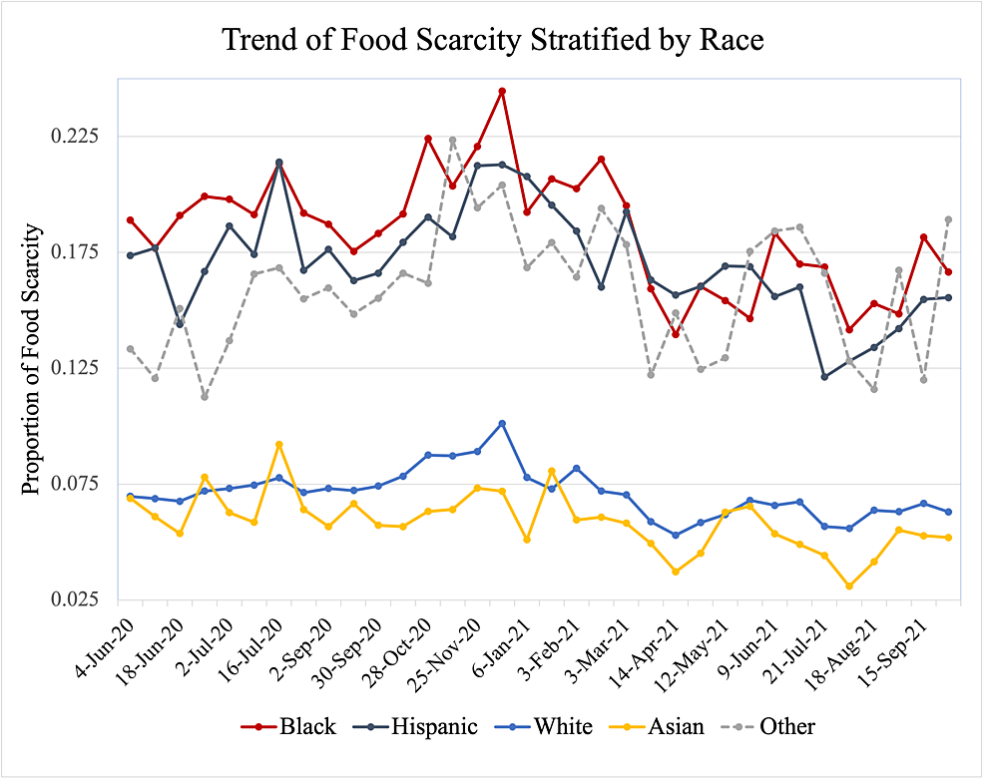
Trend of Food Scarcity Stratified by Race

**Figure 2:**
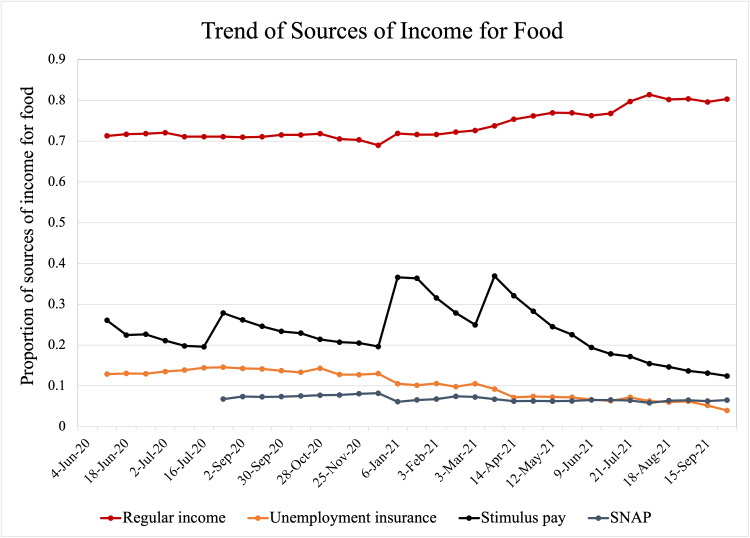
Trend of Sources of Money for Food SNAP, Supplemental Nutritional Assistance Program.

Of an estimated average of 249,546,185 US adults per survey, 62.5% were White, 17.1% Hispanic, 11.4% Black, 5.2% Asian, and 3.8% other racial minorities. Adults aged 25 to 54 years accounted for 51.6%, followed by adults aged 65 years and older (22%) and adults aged 18 to 24 years (8.7%). Females comprised 51.6%. On average during the study period, 12.1% of Hispanics, 14.0% of Blacks, and 11.3% of other racial minorities were classified as having food scarcity as compared to 5.3% of White and 3.1% of Asian adults. Time series plots suggested that the proportion of food scarcity was incremental from June to December 2020 and began decreasing after December 2020 (Figure 1). This trend was seen across all racial groups, but the gaps in food scarcity rates narrowed between Black/Hispanic and White groups. At the same point in time, regular income rates began to rise while the use of food assistance programs, unemployment insurance, and COVID-19 stimulus payments remained unchanged or reduced (Figure 2).

## Discussion

The findings of the present study showed that Black, Hispanic, and other racial minority adults were more likely to experience food insecurity and scarcity, compared to White and Asian adults during the COVID-19 pandemic in the United States. The improvement of food scarcity was observed in all racial groups after December 2020 and the disparity between the racial/ethnic minorities populations, including Blacks and Hispanics, and Whites and Asians had been narrowing since. According to our preliminary analysis, the regular income rate has been the most significant contributor as a source of money for food throughout the pandemic and its contribution has increased as a source after December 2020.

Food insecurity, defined as a condition in which households lack access to adequate food because of limited financial resources, is a leading health and nutrition issue in the United States [2]. In 2019, almost fourteen million Americans experienced food insecurity [3]. In recent years, multiple studies highlighted the importance of food insecurity as SDOH and the adverse effect of food insecurity on adults with chronic diseases such as diabetes mellitus, hypertension, hyperlipidemia, and obesity, resulting in higher mortality in cardiovascular disease [9-12]. Similarly, the burden of food insecurity has been imposed on children [13].

During the pandemic, Black and Hispanic populations experienced the worse clinical courses including higher infection and death rates from COVID-19 as well as higher rates of losing employment, income, and childcare [14,15]. Furthermore, a few reports showed that during the pandemic food insecurity worsened disproportionally in vulnerable populations such as the non-White population [15,16]. For instance, one study using HPS showed that Black, Hispanic, and Asian adults exhibited much worse mental health during the pandemic compared to before the pandemic, although there are a limited number of studies that examined the impact of food insecurity on other chronic/acute medical conditions. 

In our time series plots, we observed that food scarcity had been improving since December 2020. Regular income had been the most significant source of money for food, followed by COVID-19 stimulus payments, unemployed insurance, and the supplemental nutrition assistance program. The regular income has increased its role as a source after December 2020. These findings suggest that the observed improvement in food insecurity was not through those food assistance programs but because of the recovery of regular income, namely, the recovery of the US economy. This finding might highlight that the government would need to introduce reliefs, such that they would be designated to address the inequities more effectively. 

One of the strengths of the present study is that the study samples, respondents of the survey, represent well US population. Even though HPS is subject to potential non-response bias, the racial demographics included in the analyses were consistent with that of overall US populations. Second, we employed a time series plot analysis. The time series plots are a sequence of observations and they enable us to evaluate and visualize changes of interest over time. This helps us to assess the trend and the pattern including seasonal patterns, cyclic patterns, random variation, sudden shifts, and disproportionate effects such as "outliners". Finally, the present study is a unique study that focused on the impact of the COVID-19 pandemic on food insecurity by races and examined the trend of financial sources for food during the pandemic. 

Our preliminary study has a few limitations. First, despite the large datasets used in our analysis, several variables were unavailable in the HPS that would have been more insightful if included in the analyses. For example, the dataset does not include immigration status. The immigration status, whether they are citizen/permanent residents or not, would often determine eligibility for some benefits including the food assistance program and COVID-19 stimulus payment. This lack of stratification may underestimate the effect of food assistance as a source of money for food for vulnerable citizens and permanent residents, given the fact that the immigrants would account more for the minority group, compared to other racial groups. Second, we explored the trend of food insecurity and the source of money for food, but these analyses cannot demonstrate association or causation. Third, nonresponse biases were accounted for by HPS survey weights to some degree but cannot be excluded or further analyzed due to a lack of available data on nonrespondents by race. For example, if people with severe food scarcity were more likely to be nonresponders, we could underestimate the food scarcity rate.

## Conclusions

In conclusion, US adults/households suffered from food scarcity amid the COVID-19 pandemic but there have been improvements in food scarcity after December 2020. It is possible that the observed improvement could be linked to the recovery of the US economy during which more and more people started gaining regular income sources. However, the racial disparity remains a concern, especially for Black and Hispanic populations. Further research is needed to quantitatively assess the observed racial differences and different degrees of impacts of economic recovery on food scarcity.
